# Calcium in peroxisomes: An essential messenger in an essential cell organelle

**DOI:** 10.3389/fcell.2022.992235

**Published:** 2022-08-30

**Authors:** Yelena Sargsyan, Julia Kalinowski, Sven Thoms

**Affiliations:** ^1^ Department for Biochemistry and Molecular Medicine, Medical School EWL, Bielefeld University, Bielefeld, Germany; ^2^ Department of Child and Adolescent Health, University Medical Center, Göttingen, Germany; ^3^ German Center for Cardiovascular Research (DZHK), Partner Site Göttingen, Göttingen, Germany

**Keywords:** peroxisomes, calcium, Ca^2+^, cell organelle, cardiomyocyte, FRET sensor

## Abstract

Calcium is a central signal transduction element in biology. Peroxisomes are essential cellular organelles, yet calcium handling in peroxisomes has been contentious. Recent advances show that peroxisomes are part of calcium homeostasis in cardiac myocytes and therefore may contribute to or even shape their calcium-dependent functionality. However, the mechanisms of calcium movement between peroxisomes and other cellular sites and their mediators remain elusive. Here, we review calcium handling in peroxisomes in concert with other organelles and summarize the most recent knowledge on peroxisomal involvement in calcium dynamics with a focus on mammalian cells.

## Introduction

Calcium ions (Ca^2+^) are among the most important intracellular second messengers with essential roles in various cellular processes such as embryonic development, muscle contraction, neuron excitability, and cell death (Berridge et al., 2000; Giorgi et al., 2018). Ca^2+^ is the only form of calcium with biological relevance and no mechanisms of its degradation or synthesis are known. Ca^2+^ is biologically active by two main mechanisms: The movement of charge along electrical currents across membranes, and binding and unbinding of target proteins translocate Ca^2+^ within cells (Görlach et al., 2015). In this context, calmodulin (CaM) is of particular importance as a Ca^2+^-binding messenger protein that acts on a wide range of cellular pathways (Cheung, 1980; Kahl and Means, 2003).

Calcium signaling can be initiated by calcium influx through the plasma membrane (PM), and by efflux from the endoplasmic reticulum (ER) (or sarcoplasmic reticulum (SR) in muscle cells), the major intracellular calcium store. ER calcium is released either into the cytosol, or through specialized compartments and membrane contact sites to juxtaposed organelles (Paupe and Prudent, 2018). Calcium release from intracellular stores activates store operated calcium entry (SOCE) from the extracellular space. Cytosolic calcium is either constantly pumped back to the ER through the sarco/endoplasmic reticulum Ca^2+^-ATPase (SERCA) or exits the cell by the plasma membrane calcium ATPase (PMCA) (Raffaello et al., 2016).

Peroxisomes are membrane-bound organelles originally identified as sites for production and degradation of hydrogen peroxide, and fatty acid metabolism (Wanders and Waterham, 2006; Islinger et al., 2018). Mutations in any of the 15 genes encoding essential peroxisomal biogenesis factors (peroxins) can cause rhizomelic chondrodysplasia punctata (RCDP) or disorders of the Zellweger syndrome spectrum (ZSS), a group of rare multisystem disorders marked by peroxisomal dysfunction and concomitant metabolic abnormalities (Klouwer et al., 2015). Peroxisomes are spherical or tubular with diameters ranging from 100 nm to 1 µM (Soliman et al., 2018; Sograte-Idrissi et al., 2020). The large range is due to species differences and depends on the methods used. The smallest diameters are detected by super-resolution microscopy (Soliman et al., 2018). Peroxisomes contain over 130 proteins participating in a large variety of metabolic pathways (Wanders, 2014). Peroxisomes play a crucial role, e.g., in ether lipid and bile acid biosynthesis, the metabolism of D-amino acids, reactive oxygen species (ROS), and the degradation of purines, polyamines and L-pipecolic acid in mammals (Wanders and Waterham, 2006; Sargsyan and Thoms, 2020). Furthermore, peroxisomes cooperate with mitochondria for the efficient β-oxidation of several fatty acid species and virtually all peroxisomal metabolic pathways require intimate communication of peroxisomes with other organelles (Sargsyan and Thoms, 2020).

In electron micrographs of rodent hearts, peroxisomes are found in immediate vicinity of T-tubules and with junctional SR (Hicks and Fahimi, 1977). T-tubule and SR interaction sites are the main determinants of excitation-contraction coupling and effective Ca^2+^ handling in cardiomyocytes (CMs) (Flucher et al., 1994). The defined localization of peroxisomes at these sites suggests that Ca^2+^ may be important for peroxisomes, and that peroxisomes may take up Ca^2+^ and are part of Ca^2+^ homeostasis in CMs (Sargsyan et al., 2021).

## Calcium presence in peroxisomes

Peroxisomes are highly dynamic organelles capable of fast adaptation to nutritional and environmental changes (Islinger et al., 2012). The multiple interconnections of peroxisomal and extraperoxisomal metabolic pathways imply that peroxisomes may be involved in the regulation of cellular processes and be a part of signaling pathways (Islinger et al., 2012; Sargsyan and Thoms, 2020). Recently, peroxisomal ether lipid metabolism was found to be essential under hypoxic conditions (Jain et al., 2020). At the same time, peroxisomal ROS regulate the activity of mTOR signaling and autophagy (Zhang et al., 2013), suggesting peroxisomes are fine-tuning cellular homeostasis at different levels.

In plants, Ca^2+^-sensitive targets involved in peroxisomal metabolism have been described. In *Arabidopsis* and tobacco, ROS-scavenging efficiency increases with Ca^2+^-mediated activation of peroxisomal catalase 3 (Yang and Poovaiah, 2002; Costa et al., 2010). Furthermore, the Ca^2+^-dependent protein kinase AtCPK1 is targeted to peroxisomes (Dammann et al., 2003) and peroxisomal Ca^2+^ and CaM are essential for protein import and functionality of peroxisomal enzymes (Corpas and Barroso, 2018), including nitric oxide (NO) synthase, which, in plants, has an inducible peroxisomal isoform and is associated with pathogen defense (Barroso et al., 1999; Corpas et al., 2004).

## Peroxisomal calcium in mammalian cells

The study of purified hamster liver peroxisomes suggested that peroxisomes store Ca^2+^ and carry a vanadate-sensitive Ca^2+^-ATPase on the peroxisomal membrane (Raychaudhury et al., 2006). Drago et al. (2008) and Lasorsa et al. (2008) were the first to measure peroxisomal Ca^2+^ in intact mammalian cells. These studies gave conflicting results about the levels of Ca^2+^ in peroxisomes and the kinetics of peroxisomal Ca^2+^ dynamics. Lasorsa et al. (2008) did not find Ca^2+^-ATPase activity in resting peroxisomes. Using an aequorin sensor, they concluded that peroxisomal Ca^2+^ concentration in steady state is around 20-fold higher than in the cytosol, rise up to 50–100 µM depending on the cell type and reach 70 µM in HeLa cells. On the other side, Drago et al. (2008) showed that peroxisomal Ca^2+^ levels are similar to cytosolic Ca^2+^ and rise slowly when the concentration of the latter rises. Differences in measurement techniques and biophysical properties of the sensors can partially explain these contradicting results (Costa et al., 2013).

In our recent study, three genetically encoded ratiometric Ca^2+^ indicators covering a broad Ca^2+^ sensitivity range—K_d_ 0.6, 1.7, and 60 µM—were used to reassess the results of the aforementioned papers (Sargsyan et al., 2021). D1cpV-px with the highest K_d_ had the lowest dynamic range and only minimal calcium-dependent increase in fluorescence resonance energy transfer (FRET) could be detected upon maximal stimulation. The response of D3cpV-px (FRET sensor) and pericam-px (ratiometric sensor) were comparable and were not saturated, suggesting that peroxisomal Ca^2+^ levels are in the optimal detection range of these sensors (Sargsyan et al., 2021). Pericam as a classical EYFP-based sensor may be pH-sensitive in an acidic environment (Nagai et al., 2001). We did not detect signal changes of YFP variants that could be attributed to Ca^2+^-independent changes of the sensor, suggesting measurements with the more pH-sensitive pericam-px are also reliable.

Parts of the results of our work are based on measurements with the same sensor (D3cpV-KVK-SKL) as Drago et al. (2008), the only difference is a stronger PTS1 (peroxisomal targeting signal 1) signal in D3cpV-px. In this manner we could overcome the problem of unspecific targeting of the sensor described by Drago et al. (2008), which these authors solved by adding a linker before the PTS1 tripeptide.

**FIGURE 1:**
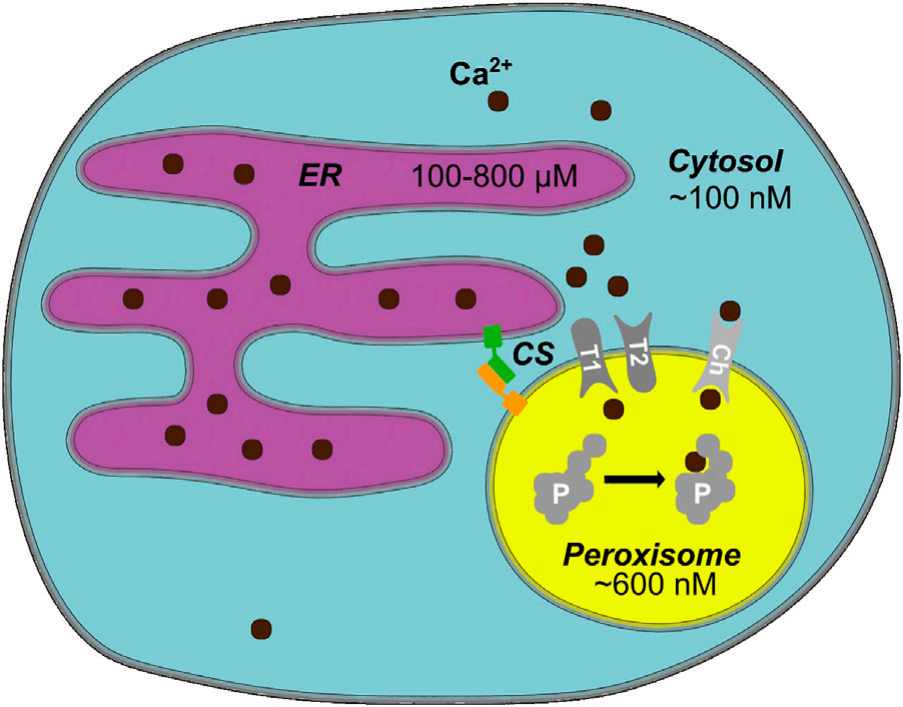
Peroxisomal Calcium—an Overview. In mammalian cells, cytosolic Ca^2+^ levels are around 100 nM, whereas the endoplasmic reticulum (ER) as a main cellular Ca^2+^ store has several hundred micromolar Ca^2+^ (Samtleben et al., 2013). An average peroxisome contains around 600 nM Ca^2+^ (Sargsyan et al., 2021). Ca^2+^ likely enters peroxisomes either through a channel/pore or a transporter (Ch). The entry and exit mechanism of Ca^2+^, however, may differ (T1/T2 hypothetical importer/exporter). Known protein tethers form contact sites (CS) between peroxisomes and the ER, potentially create microdomains that facilitate the exchange of Ca^2+^. Ca^2+^ may bind to a yet unknown intraperoxisomal protein (P) or membrane protein and affect its function, e.g., by inducing a conformational change when bound to Ca^2+^. Hypothetical elements of the model (channel, transporter, intraperoxisomal Ca^2+^-sensitive protein) are shown in gray.

In HeLa cells, we found basal peroxisomal Ca^2+^ levels with a mean value of 600 nM and increase upon stimulation up to 2.4 µM (Sargsyan et al., 2021). Of note, 7% of the analyzed cells had basal peroxisomal Ca^2+^ higher than 1 μM, which would be over 10-fold higher than the expected cytosolic level and would partially correspond to the findings of Lasorsa et al. (2008). Upon stimulation, again some rare cells showed an extremely high increase of peroxisomal Ca^2+^ up to 6.5 µM and higher. The absence of correlation between the high maximal response and basal Ca^2+^ values speaks against the hypothesis that peroxisomes have a strictly limited Ca^2+^ uptake capacity and may rather imply that Ca^2+^ increase in peroxisomes highly depends on the cell state and current cellular needs. Our findings are integrated in an updated overview of organellar calcium concentrations (Figure 1).

## Peroxisomal calcium in cardiomyocytes

The role of Ca^2+^ is even broader for CMs than in other tissues. Here, Ca^2+^ interconnects the electrical stimulation of cardiac myocytes and their contraction—a process termed excitation-contraction coupling (Bers, 2008). The specific crucial molecular players of Ca^2+^ handling in CMs are ryanodine receptors (RyR) on the SR, voltage-operated Ca^2+^ channels in the T-tubules and Na^+^/Ca^2+^-exchanger on the plasma membrane. Strict control of cellular Ca^2+^ levels is of particular importance in cardiomyocytes. Ca^2+^ overload in CMs results in malfunction of the heart by affecting both the electrical and contractile properties of cardiomyocytes (Vassalle and Lin, 2004). Electrical abnormalities of CMs present as arrhythmias with varying severity from relatively harmless to life-threatening (Vassalle and Lin, 2004; Landstrom et al., 2017).

The localization of peroxisomes in proximity to the T-tubular system and SR in cardiac myocytes (Hicks and Fahimi, 1977) hinted at a role for peroxisomes in Ca^2+^ handling. Intracellular store-depletion by the activation of RyRs on the SR through Ca^2+^ from the T-tubule localized L-type Ca^2+^ channel (LTCC, Ca^2+^-induced Ca^2+^-release) is the main source of Ca^2+^ increase in CMs in the excitation-contraction coupling (Bers and Perez-Reyes, 1999). Using chemical stimulation, we have shown that Ca^2+^ enters peroxisomes upon intracellular Ca^2+^-store depletion in neonatal rat CMs (NRCMs) and human induced pluripotent stem cell-derived CMs (hiPSC-CMs) (Sargsyan et al., 2021). We hypothesized that cardiac peroxisomes take up Ca^2+^ on a beat-to-beat basis. Indeed, we showed that upon electrical field stimulation with 1 Hz frequency, peroxisomes in NRCM take up Ca^2+^ in beat-to-beat manner (Sargsyan et al., 2021).

Peroxisomes in hiPSC-CMs occasionally localize in vicinity of ER protein RyR2 and rarely to T-tubular system and LTCC (Sargsyan et al., 2021). The striation pattern is not well-developed in hiPSC-CMs and therefore the relative localization of RyR2, LTCC, and peroxisomes in hiPSC-CMs may differ from that in CMs in the beating heart. It is known from monkey kidney fibroblast-like COS-7 cell line that over 90% of peroxisomes are in contact with the ER (Valm et al., 2017). ER-peroxisome contact is dependent on ACBD4/5-VAPB, and on Miro1v4-VPS13D-VAP (Costello et al., 2017a, 2017b; Hua et al., 2017; Guillén-Samander et al., 2021). However, it is not known whether membrane contact sites are relevant for Ca^2+^ entry to peroxisomes, if there are tissue or cell type-specific molecular composition and/or abundance of contact sites, and which of these are relevant for CMs.

As peroxisomes take up Ca^2+^ from the SR and localize in the proximity of RyR receptors in CMs, it is plausible that peroxisomes may contribute to or even be essential for normal excitation-contraction coupling. This hypothesis is supported by the fact that patients with mild forms of ZSS occasionally present with cardiac arrhythmias that may become the cause of lethal outcome (Wanders and Komen, 2007). The metabolic role of peroxisomes could be the reason for these arrhythmias (Colasante et al., 2015). However, a direct contribution to efficient Ca^2+^ handling by the peroxisomes is another plausible reason.

## Candidates of peroxisomal calcium channels

Although peroxisomal Ca^2+^ changes largely follow cytosolic Ca^2+^, our experiments with maximal Ca^2+^ mobilization through ionophore addition showed a slower Ca^2+^ increase and even slower decline in peroxisomes compared to the cytosol (Sargsyan et al., 2021). The slow but constant increase in peroxisomal Ca^2+^ when the cytosolic Ca^2+^ rises suggests that the transfer mechanism may have limited capacity and can be saturated. Potential peroxisomal Ca^2+^ channels and pores are PEX11 (Mindthoff et al., 2016), PXMP2 (Rokka et al., 2009), PMP34 (Wylin et al., 1998), or any other peroxisomal membrane protein with a transport function (Chornyi et al., 2020). PEX11 and PXMP2 are reported to be unspecific peroxisomal channel-forming proteins with permeability to small solutes in *in vitro* experiments on artificial membranes (Rokka et al., 2009; Mindthoff et al., 2016). PMP34 has been suggested as a coenzyme A, FAD, and NAD^+^ transporter across peroxisomal membranes using liposomes with reconstituted recombinant protein (Agrimi et al., 2012). An *in vivo* study investigating the channel function of PXMP2 and PEX11 examined their role in hydrogen peroxide transport across peroxisomal membrane (Lismont et al., 2019). Judging from the molecular weight of the hydrogen peroxide it could freely pass through both PXMP2 and PEX11. Nonetheless, a fluorescent biosensor for H_2_O_2_ in PXMP2- and/or PEX11-deficient cells, showed that neither PXMP2 nor PEX11 are essential for H_2_O_2_ trafficking across the peroxisomal membrane (Lismont et al., 2019). Altogether, this suggests that the search for peroxisomal Ca^2+^ transport machinery may present a challenging task.

## Biological relevance of peroxisomal calcium

Highly localized calcium dynamics play a central role in controlling cellular processes. At the same time, excessive increase of intracellular Ca^2+^ to levels that cannot be handled by the cell is known as Ca^2+^ overload and can have detrimental consequences (Vassalle and Lin, 2004). For example, mitochondrial calcium stimulates energy gain from Krebs cycle and respiratory chain but can also induce cell death in case of mitochondrial Ca^2+^ overload (Görlach et al., 2015). Thus, the subcellular sequestration of Ca^2+^ in different compartments is vital for the regulation of physiological processes.

The interplay of redox and Ca^2+^ signaling is well described for mitochondria, where Ca^2+^-dependent opening of calcium channels is regulated by interaction with the oxidoreductase Mia40 (Petrungaro et al., 2015). Additionally, metabolic processes also regulate Ca^2+^ uptake by mitochondria (Nemani et al., 2018). Whether redox or metabolic processes influence peroxisomal Ca^2+^ is not known. There is also no consensus about the drivers of Ca^2+^ transport to peroxisomes and the role of known ion transporters like Ca^2+^/H^+^ antiporter, Ca^2+^/Na^+^ exchanger, and V-ATPase for peroxisomal Ca^2+^ levels (Drago et al., 2008; Lasorsa et al., 2008).

Presently, in contrast to plant catalases and kinases (Yang and Poovaiah, 2002; Dammann et al., 2003), no mammalian peroxisomal enzymes are known to bind Ca^2+^. Weber et al. (1997) suggested the presence of a Ca^2+^-dependent mitochondrial solute carrier *Efinal* (gene *SLC25A24*) also on peroxisomes based on immunoelectron microscopy in rabbit small intestinal tissue. Along with peroxisomal malate dehydrogenase and lactate dehydrogenase (Schueren et al., 2014; Hofhuis et al., 2016; Schueren and Thoms, 2016), they would serve as crucial components of malate and lactate shuttles across peroxisomal membranes for reduction equivalent reoxidation (McClelland et al., 2003). However, in human cell lines, *Efinal* homologues—identified as members of the short calcium-binding mitochondrial carriers (SCaMC) protein subfamily—were found exclusively in mitochondria (del Arco and Satrústegui, 2004).

Direct evidence for a functional role of intra-peroxisomal Ca^2+^ is still missing. For the mammalian peroxisome, however, Ca^2+^ appears to be important. Particularly, Ca^2+^ channel blockers nifedipine, diltiazem and nicardipine suppress peroxisomal fatty acid oxidation enzymes and peroxisome proliferation (Watanabe and Suga, 1988; Itoga et al., 1990; Zhang et al., 1996). These findings might be due to direct regulation of a peroxisomal enzyme through Ca^2+^ or by indirect regulation of peroxisomal functions by extraperoxisomal Ca^2+^.

In case of the latter, peroxisomal membrane protein Miro1v4 could be a potential target. Miro1v4 is a peroxisomal variant of mitochondrial the Miro1 protein that form ER contact sites through VPS13D to exchange lipids and has been shown to mediate the linkage of mitochondria to motor proteins in a calcium-dependent manner (MacAskill et al., 2009). Miro1v4 has two potentially Ca^2+^ binding EF-hands which are essential for Miro-VPS13D interaction on peroxisome-ER contact sites (Guillén-Samander et al., 2021). Whether Ca^2+^ binding really regulates peroxisome-ER contact was not studied experimentally. Still, no Ca^2+^-dependent interaction of the mitochondrial-ER contact site mediated by Miro-VPS13D could be found in experiments (Guillén-Samander et al., 2021). Other lipid transfer mechanisms, like the vesicle-based lipid exchange mediated by synaptotagmin, have been shown to be Ca^2+^-dependent (Yu et al., 2016). Similarly to the findings for mitochondria, peroxisomal Miro isoforms have been implicated in peroxisome motility (Castro and Schrader, 2018) although there is no evidence on involvement of Ca^2+^ in peroxisome motility in this context.

The role of peroxisomal Ca^2+^ might not necessarily be the regulation of peroxisomal processes. Drago et al. (2008) have suggested that peroxisomes may serve as an additional cytosolic Ca^2+^ buffer compartment. This idea is supported by the fact that peroxisomal Ca^2+^ rises after ER-store depletion in case of knockdown of mitochondrial calcium uniporter (MCU) beyond its initial maximum (Sargsyan et al., 2021). This suggests that at least in some situations of cellular Ca^2+^ overload peroxisomes may take up more Ca^2+^ than under near-physiological standard conditions, therefore buffering potentially deleterious effects of excess Ca^2+^ on the cell. Consequently, the protective effect of peroxisomal Ca^2+^ uptake may be necessary only in some special situations such as Ca^2+^ overload.

As Ca^2+^ concentration in the peroxisome is higher than in the cytosol, peroxisomes may also in extreme situations play a role of additional Ca^2+^ store for the cytosol. The buffering function of peroxisomes may be protective in some cases of arrhythmia, such as catecholaminergic polymorphic ventricular tachycardia, when increased predisposition to inadequate SR Ca^2+^ release events (so called Ca^2+^ sparks) occur. On the other hand, the absence of peroxisomes may contribute to cardiac pathogenesis and promote the development or extent of arrhythmias.

## Conclusion

Ca^2+^ enters peroxisomes of non-excitable and excitable mammalian cells upon near-physiological electrical and chemical stimulation. Peroxisomal Ca^2+^ handling presents an exciting research area with many open questions: For example, the existence of Ca^2+^-dependent transporters in peroxisomes as suggested (Weber et al., 1997) is still unclear. Similarly, Ca^2+^-sensitive targets in the mammalian peroxisome, as known for the plant peroxisome, may yet be identified. Furthermore, it is conceivable that peroxisomal Ca^2+^ homeostasis may be important in the absence of luminal Ca^2+^ binding proteins, by buffering local calcium. In excitable cells, peroxisomal Ca^2+^ dynamics might be of special importance, also with regard to their pathophysiology. The cellular function of peroxisomal Ca^2+^ and the role of peroxisomal Ca^2+^ in pathology remain to be studied further.
